# Evolution of ferromagnetic cluster in perovskite La_0.88_Sr_0.12_MnO_3_ nanocrystalline detected by EPR spectrum

**DOI:** 10.1038/s41598-024-63512-y

**Published:** 2024-06-05

**Authors:** Shaozhen Li, Lisha Xu, Chao Fu, Mengqiu zheng, Wei Tong, Jiyu Fan

**Affiliations:** 1 School of Electrical and Electronic Engineering, Wuhan Polytechnic University, Wuhan, 430023 Hubei China; 2 School of Physics and Institute for Quantum Materials, Hubei Polytechnic University, Huangshi, 435003 China; 3 Anhui Key Laboratory of Condensed Matter Physics at Extreme Conditions, High Magnetic Field Laboratory, Hefei Institutes of Physical Science, Chinese Academy of Sciences, Hefei, 230031 China; 4 Department of Applied Physics, Nanjing University of Aeronautics and Astronautics, Nanjing, 210016 China

**Keywords:** La_0.88_Sr_0.12_MnO_3_ nanocrystalline, Griffiths phase, EPR spectrum, Ferromagnetic clusters, Materials science, Nanoscience and technology, Physics

## Abstract

Electron paramagnetic resonance (EPR) studies were performed on La_0.88_Sr_0.12_MnO_3_ (LSMO) nanocrystalline together with the measurement of its magnetization. Various spectrum parameters including line width, effective *g*-value and double-integrated intensities have been analyzed in detail. We found nonlinear behavior occurred in the inverse susceptibility far above the Curie temperature *T*_*C*,_ indicating short-range ferromagnetic (FM) clusters and Griffiths-like phase behavior in the paramagnetic (PM) phase. Based on the variation of EPR spectra, except for a typical PM resonance peak, an extra resonance signal was observed in the lower field region and developed as temperature decreased from 320 K to 110 K, which gave a direct evidence of the existence of FM cluster in the PM region of LSMO nanocrystalline. We proposed that the appearance of the Griffiths phase was due to the short FM correlation in the PM regime enhanced by surface spin ordering.

## Introduction

Griffiths singularities have been reported in many complex magnetic materials including perovskite manganites R_1*−x*_A_*x*_MnO_3_ (where R is a rare-earth element such as La, Pr, Sm and A are divalent alkaline-earth element Ca, Sr and Ba)^1–5^, rare-earth intermetallic system and Heusler alloy^6–9^. Generally, the Griffiths singularity accounts for a magnetic system with a random distribution of magnetic interactions given by disordered sets in such a lattice or valence/spin state. In the temperature region *T*_*C*_ < *T* < *T*_*G*_ (*T*_*G*_ is the Griffiths-like temperature scale), the conventional PM phase and FM phase coexist in materials and form a magnetic disordered system. In this condition, a FM cluster usually occurs at temperature higher than Curie temperature (*T*_*C*_). Bustemrgy et al.^10,11^ have further more argued that the coexistence of two competing ordered phases stabilized and enhanced the Griffiths-like effects. Therefore, the magnetic anisotropy and the intrinsic disorder which are in inherent in a magnetic system play a key role for the Griffiths phase.

In this work, the EPR spectrum and magnetic susceptibility were measured to detect the Griffiths phase and FM clusters in the LSMO nanocrystalline. EPR spectroscopy is a powerful probe for studying spin dynamics, magnetic correlation and phase coexistence in perovskite manganites on a microscopic level. In previous studies, the phase coexistence, magnetic anisotropy, and electronic phase separation behavior have been detected directly with EPR spectra^12–15^. For instance, Deisenhofer et al.^15^ observed the FMR signals in La_1*−x*_Sr_*x*_MnO_3_ single crystal at an extended temperature range above *T*_*C*_. As well as in La_1*−x*_Ca_*x*_MnO_3_ (0 < *x* ≤ 0.23)^14^ and La_0*.*6_Er_0*.*1_Sr_0*.*3_MnO_3_ perovskite polycrystal^16^, the EPR spectra also shows a coexistence of PM and FM microdomains. In these magnetic materials, an extra resonance peak is highly visible in the low field region from EPR spectra. Moreover, this peak strongly depends on the change of temperature, which is generally referred to the fingerprint of FM microdomain or clusters^2,5,14,17,18^. For bulk samples, the double peaks of a Lorentzian curve in EPR spectroscopy can be attributed to the local chemical disorder and complex electronic/magnetic interaction. However, when the sample size of perovskite manganites was decreased to nanoscale, the increased surface to body ratio increases the magnetic inhomogeneity and suppresses the FM-type signal in EPR spectra^2^.

Here, we report a stable Griffiths phase in LSMO nanocrystalline detected with EPR spectra. The EPR spectra show an extra peak from 360 K to 200 K, indicating that the PM phase and FM microdomain/clusters coexist at high temperature. This phenomenon is very different from the observation reported by Rozenberg et al.^2^, where the FM-type signal is suppressed as the size of LSMO is decreased to nanoscale. Generally, the nanocrystalline has a better chemical and structural homogeneity than polycrystalline, but an increase of surface spin disorder weakens the inner magnetic coupling. By analyzing the temperature dependence of various spectrum parameters, including double integrated intensities, peak-to-peak linewidth ∆H*pp*, and effective *g*-factor, we propose that the surface spin ordering can facilitate the establishment of short FM correlation in the PM phase.

## Experimental procedure

The nanosized La_0*.*88_Sr_0*.*12_MO_3_ were synthesized by the sol–gel method with high-purity La_2_O_3_, SrCO_3_, and MnCO_3_ as starting materials (all 99.99% from Alfa Aesar; using the general method), all materials were converted into their nitrates by adding nitric acid with a molar ratio of La/Sr/Mn = 0.88:0.12:1. Secondly, when the precursor metal nitrate solutions were formed, the citric acid was added as a polymerizing agent. Next, the gel was obtained after gradual evaporation of the solvent at 75 °C for 10 h. After that, the gel was dried at 100 °C for 24 h to remove and then to remove and decompose nitrates, so it was preheated to form a black porous powder at 400 °C for 6 h. Finally, the precursory powder was annealed at 800 °C for 4 h to produce nanoparticles with an average particle size. The structure and the phase purity of the prepared sample were checked by X-ray diffraction (XRD), using Cu-K*α*1 radiation (D5000 diffractometer) at room temperature. The particle sizes and morphology were determined by field emission scanning electron microscopy (FE-SEM, JEOL-6700F).

The magnetic measurements were carried out using a commercial superconducting quantum interference device magnetic property measurement system (SQUID VSM-Quantum Design, Inc.). The electron paramagnetic resonance (EPR) measurement of the powder sample was performed at selected temperatures using a Bruker EMX-plus model spectrometer with a heater operating at X-band frequencies.

## Results

**Figure 1:**
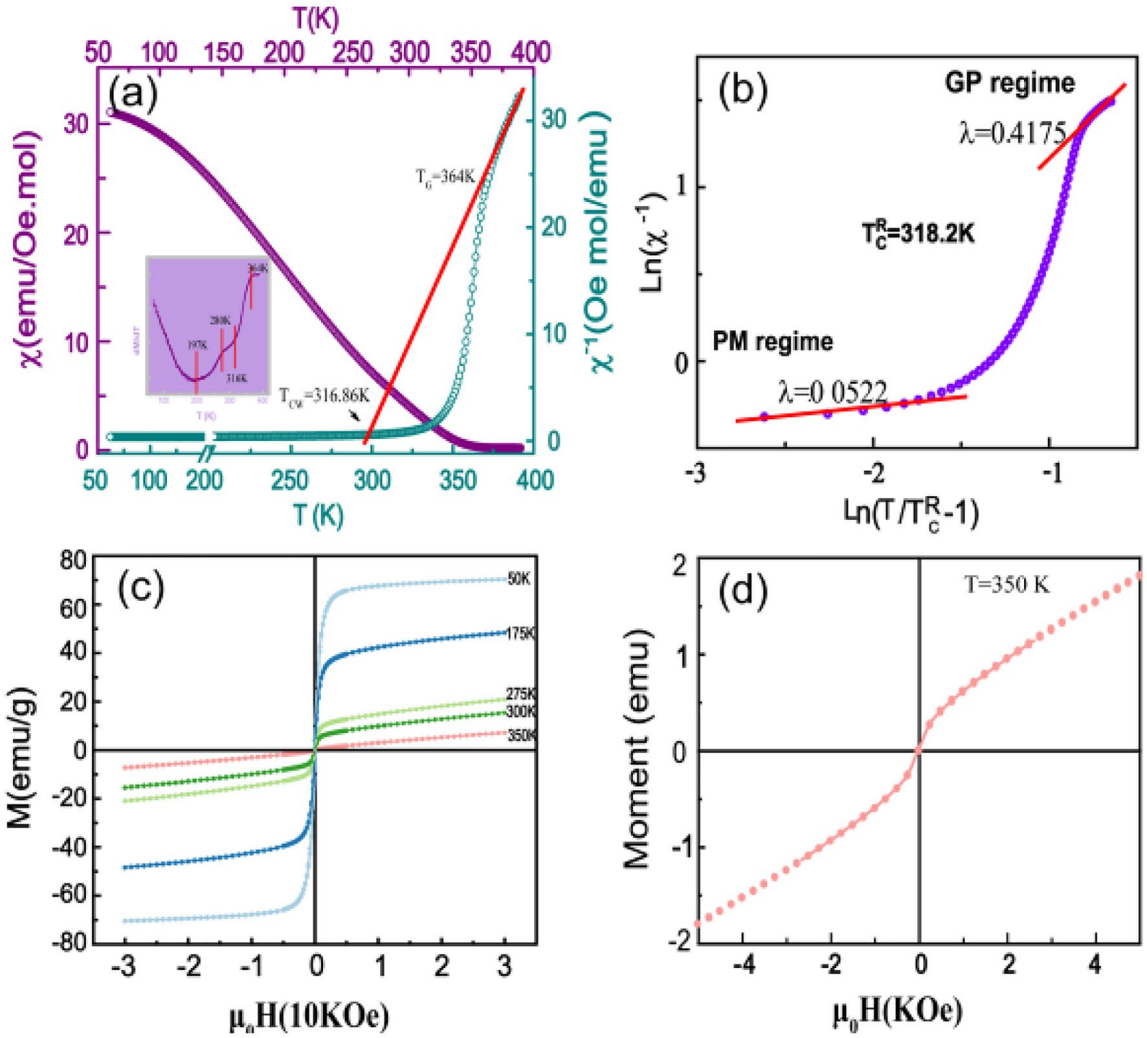
(Left axis) (**a**) Temperature dependence of magnetization measured at H = 100 Oe. Right axes Inverse susceptibility as a function of temperature for La_0*.*88_Sr_0*.*12_MnO_3_. The dash lines represent the fitting data according to the Curie–Weiss law and the solid line is the fitting result following the Griffiths model. (**b**) log–log scale, and the red line is the fitted data of formula. (**c**) Magnetic hysteresis loops of La_0.88_ Sr_0.12_ MnO_3_ at 50 K, 175 K, 275 K, 300 K, and 350 K from 3 T to − 3 T. (**d**) The Magnetic hysteresis loop at 350 K.

**Figure 2:**
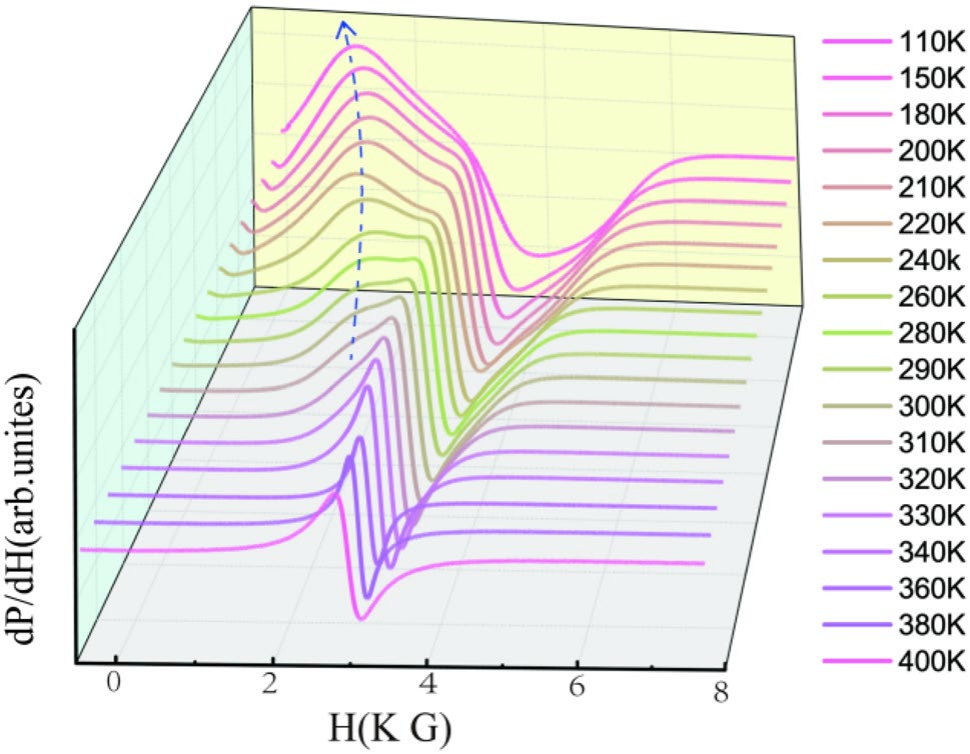
Series of EPR resonance spectra (dP/dH) from a temperature of T = 110 K to T = 440 K with 3D plotting.

The LSMO structure and phase purity were detected by the X-ray diffraction patterns. The diffraction pattern indicates that the sample is a single phase with an orthorhombic (*Pnma*) structure without any impurities. Based on these XRD patterns, Rietveld refinements were carried out using a hexagonal lattice type with *P63-mmc* space group. The calculated patterns are in agreement with the results of diffraction data as shown in Fig. 1S. The XRD peaks are broad with large full width at half maximum (FWHM), indicating the formation of LSMO nanocrystalline. The size of LSMO particles is homogeneous and the average diameter is about 30 nm estimated through FESEM in Fig. 2S. Moreover, the average particle size *d* can be calculated with the Scherrer formula *d* = *kλ/β*cos*θ,* where *k* = 0.89 is the particle shape factor, *λ* = 0.15406 nm is the wavelength of Cu K*α* radiation, and then *β* and *θ* are the full width at half maximum of the XRD (110) peak and the diffraction angle of this peak, respectively. The calculated average particle size is about 25.6 nm, which is similar with the results in FESEM micrograph.

Figure 1a presents the temperature dependence of magnetic susceptibility (*χ* − *T*) measured in a magnetic field of 100 Oe (purple, left hand axis) and inverse DC magnetic susceptibility *χ*^*−*1^ (dark cyan) with the fitting results (red line, right) according to the Curie–Weiss law,

1 $$\chi = C/(T - T_{CW} )$$

where *C* is the Curie constant defined as:* C* = 3 *N*_*A*_ * k_*AB*_ (*µ*^*exp*^_*eff*_) ^2^ (N_*A*_ = 6.023 * 10^23^ mol^*−*1^ is Avogadros, number *k*_*B*_ = 1.38016 * 10^*−*16^ J K^*−*1^ is Boltzmann’s constant, *µ*^*exp*^_*eff*_ is the experimental effective moment and *T*_*CW*_ is the Weiss temperature). At *T* > 364 K, the behavior of *χ*^*−*1^ presents a straight line. Clearly, in the conventional PM region, a linear fit to high temperature yields the Curie constant (*C* = 3.636 emu K/mole Oe) and a positive Curie–Weiss temperature *T*_*CW*_ = 316.86 K; At *T* < 364 K, a sharp decrease behavior of *χ*^*−*1^ was observed. The deviation of the inverse susceptibility from the high-temperature straight line indicates the onset of a new magnetic interaction between magnetic moments. This anomalous behavior implies that the Griffiths singularity possibly appears because this intermediate phase transition gives rise to a downward curvature in inverse susceptibility curves above *T*_*C*_ = 197 K^19^. The Griffiths singularity can be solely characterized by a susceptibility exponent *χ*(T)^*−*1^ lower than unity: a power-law relationship^20,21^,

2 $$\chi (T)^{{ - }{1}} \propto (T - T^{R}_{C} )^{{{1} - \lambda }} ,$$

where 0 ≤ *λ* < 1 and *T*^*R*^_*C*_ are associated with the critical temperature of random FM phase where the susceptibility tends to diverge. In order to obtain an accurate value of *λ*, *χ*(*T*)^*−*1^ versus (*T* − *T*^*R*^_*C*_)^1*−λ*^ is plotted on a log–log scale and the slope of the fitting line in the GP and PM regime gives *λ*_*GP*_ and *λ*_*PM*_, respectively. In general, an accurate *T*^*R*^_*C*_ can lead to a physical fitting and determination of *λ* for the Griffiths phase formation. The solid line in Fig. 1b is the fitting result and the corresponding fitting parameters *λ* and *T*^*R*^_*C*_ are 0.4175 and 318.2 K, respectively. Clearly, these fitting parameters produce a good fit to the data. The Griffiths-like phase that occurred in this sample is related to the local FM fluctuations, which might be ascribed to the random spatial variation in magnetic exchange interactions.

The inset of Fig. 1a plots the temperature dependence of the d*M*/d*T* curve. There are four characteristic temperatures^22^: *T* = 197 K, 280 K, 316 K and 367 K. Meanwhile, *T* = 197 K is the minimum in the d*χ*/d*T* curve, generally defined as the Curie temperature *T*_*C*_. The temperature range of 280 K < *T* < 316 K is an unusual plateau. For most magnetic systems in a pure PM state, their d*M*/d*T* curves always show a continuous variation in the temperature region *T* > *T*_*C*_. Here, the appearance of a unusual plateau implies the possible existence of a FM cluster, spin fluctuation, and quenched disorder. At *T* > 367 K, the magnetization remains almost unchanged with the decrease in temperature, corresponding to a pure PM state. On the other hand, the average particle size is calculated about 25.6 nm, which probably causes the appearance of supermagnetic property in LSMO. Meanwhile, from the Fig. 1c, we can find that all of isothermal magnetization curves at high temperature region are separate instead of overlapping state. Therefore, the supermagnetic behavior can been excluded. In order to clarify the magnetic characteristics at these four temperatures, the isothermal magnetizations *M(H)* are measured at five selected temperatures (*T* = 50 K, 175 K, 275 K, 300 K, and 350 K) as shown in Fig. 1c. Figure 1d shows the magnified magnetic hysteresis loop of *T* = 350 K. Obviously, the loop shows a perceivable inflection, indicating the existence of FM behavior. At *T* = 300 K, between 280 K and 316 K, its magnetic hysteresis loop shows obvious FM behaviors, which means that the localized FM clusters have been formed^6^. At *T* = 50 K and *T* = 175 K, lower than the Curie temperature, its magnetic hysteresis loop implies a pure FM state. Therefore, the unusual plateau in the d*M*/d*T* curve can be originated from the localized FM cluster, which generates the Griffiths phase in LSMO nanograins.

In order to confirm the GP in LSMO nanocrystalline, the EPR spectra were measured from 110 K to 440 K. As introduced in the previous section, EPR spectroscopy has been widely used to probe spin–lattice coupling and spin–spin relaxation in perovskite manganites^23–27^. Figure 2 presents a series of EPR resonance spectra (dP/dH) with 3D plotting. When the temperature decreases from 320 K, it can be seen that the EPR spectra not only consists of the initial PM resonance (PMR) signal due to the majority of Mn^3+^ and Mn^4+^ spins, but also exhibits a nascent FM resonance (FMR) signal at a lower resonance region. The PMR and FMR signals coexist in a very wide temperature range of 150 K–320 K, but the Curie temperature *T*_*C*_ is 197 K. That means the PM resonance does not diminish even as the temperature below *T*_*C*_. In general, the low field peak was referred to as the fingerprint of FM microdomains or clusters, indicating that two different magnetic phases coexist in the system^2,14,28^.

**Figure 3:**
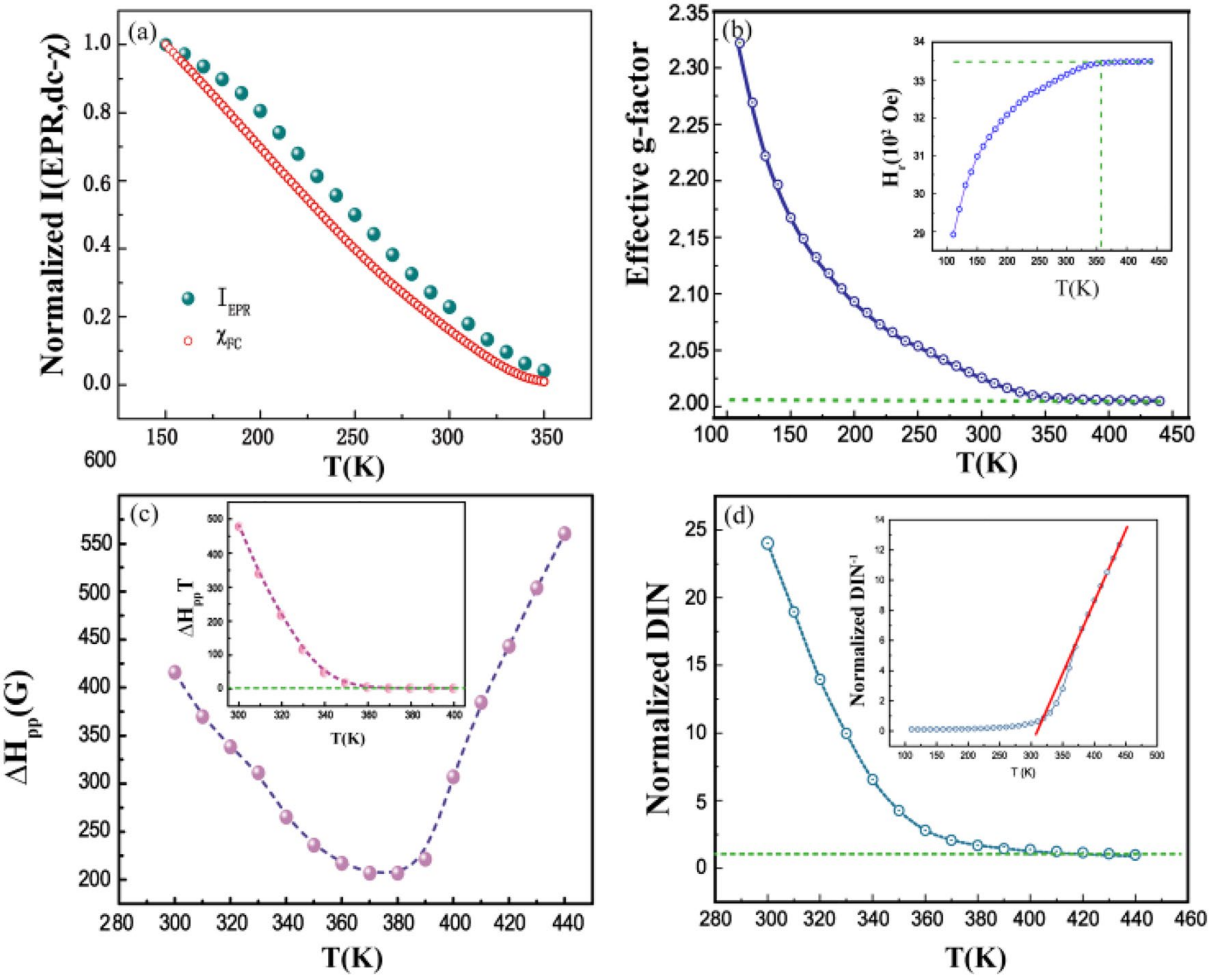
Temperature dependence of (**a**) EPR resonance intensity (I*EPR*), (**b**) Effective *g*-factor, insert shows the temperature dependence of resonance field *Hr*, (**c**) peak-to-peak linewidth ∆H*PP*, inset shows the temperature dependence of product ∆H*PP* *T*χ*(T)* and (**d**) double-integrated intensity (DIN), insert plots the inverse of DIN.

In fact, based on the previous analysis, *T* = 320 K is 3.14 K higher than the Curie–Weiss temperature T_*CW*_ = 316.86 K, and much higher than the Curie temperature, it should be clear that some localized FM interactions have generated in the PM state. In previous research, some similar EPR spectra were detected in La_1*−x*_Ca_*x*_MnO^14^. Dagotto et al.^10,11^ proposed that the perovskite manganites would be essentially suitable for EPR due to the intrinsically inhomogeneous and quenched disorder in these correlated electronic oxides. Therefore, the FM order would derive from the areas where the concentration of Sr^2+^ on A-site sublattice is larger than others' and closer to the optimal Sr^2+^ concentration (33%) for the FM double exchange effect. Here, the current LSMO nanocrystal was fabricated by a sol–gel method, thus the chemical disorder cannot be avoided. Therefore, some localized FM interactions have formed in advance before the appearance of PM–FM transition. Moreover, below *T* < *T*_*C*_ (197 K), even at *T* = 110 K, a PM peak is still observed. Even though it’s not obvious, this reason for this phenomenon is from much more the PM–FM phase transition in LSMO nanocrystal so that some PM clusters still embed in the FM state and their variations are shown in Fig. 3.

The EPR resonance intensity (*I*_*EPR*_) is an important parameter to identify the nature of the magnetic ions contribution to the resonant entities^29^. For further understanding the EPR properties in this sample, some relevant parameters including *I*_*EPR*_, resonance field position (*H*_r_), effective *g*-factor, and double-integrated intensity (DIN) are deduced from Fig. 2. As show in the Fig. 3a, the resonance intensity *I(T)* follows the DC magnetic susceptibility *χ*_*dc*_ closely, indicating that the magnetic variations detected from the EPR spectra are completely consistent with the results measured from DC magnetization^14,30^. Furthermore, it can be found that the variation of *I*_*EPR*_ completely coincides with *χ*_*dc*_ near *T* = 364 K, whereas the variation of *I*_*EPR*_ has gradually deviated from the *χ*_*dc*_ when the temperature is far from *T* = 364 K. On the other hand, a small concentration of FM inhomogeneity has been traced in the PM regime, indicating the Griffiths phase generates at *T* = 364 K.

Figure 3b summarizes the value of the *g*-factor, and the inset presents the temperature dependence of the resonance field position (*Hr*). The *Hr* varies weakly above 364 K but has a large dependence on the temperature below 364 K. The effective *g*-factor can be calculated from the resonance field formula *g* = *hν/µ*_*B*_*H*_*res*_ (*h* is the Plank constant; *ν* is the frequency of the microwave; *µ*_*B*_ is the Bohr magneton). The *g* value is close to 2 and then increases as the temperature decreases from *T* = 360 K, which coincides with the resonance field (*H*_*r*_). A similar variation of *g*-factor was also reported previously in Nd_0*.*5_Sr_0*.*5_MnO_3_ and Pr_0*.*5_Sr_0*.*5_MnO_3_^31,32^. Generally, in the PM region, the value of the *g*-factor is close to 2, which is typical for Mn^4+^ in the [MnO_6_]O^2*−*^ octahedron coordination indicating that the majority of *e*_*g*_ electrons leave Mn^3+^ ions and become either itinerant or localized outside the Mn^4+^ ions. As the temperature decreases from 360 K, the *g*-factor value shows a noticeable increase, implying the development of orbital ordering. This phenomenon of the *g*-factor can be attributed to the orbit ordering which influences the crystal field splitting and hence leads to an increase in the g value. However, the *g*-factor value increases from *T* = 360 K, much above *T*_*C*_, and this result indicates the FM ordering has been formed and the Griffiths Phase appears.

Figure 3c shows the temperature dependence of the EPR linewidth *∆H*_*PP*_ in the PM regime from 300 K to 440 K, which is defined as the width between the highest and the lowest points in the EPR spectrum. Generally, the EPR linewidth *∆H*_*PP*_ shows the quasilinear behavior near *T*_*C*_ and a minimum value around *T*_*C*_^33^. However, in Fig. 3c, the minimum temperature is at 370 K( ≈ 1.9*T*_*C*_), very close to the onset of downward deviation in the inverse susceptibility 1/*χ*(T) curve (*T*_*G*_ = 364 K). This result furthermore demonstrated the presence of FM clusters in the PM regime at *T*_*C*_ < *T* < *T*_*G*_, consistent with the DC magnetic measurement in Fig. 2.

The broadening of the EPR linewidth *∆H*_*PP*_ is more clear than in the previous studies. The EPR linewidth can be described^18^:

3 $$\Delta H_{PP} (T) = \Delta H_{PP} (\infty )[\chi_{0} (T)/\chi (T)]$$

where *χ*_0_ = (C/T) is the free-ion susceptibility with the Curie constant(C), *χ*(T) is the DC magnetic susceptibility of the couple system, and *∆Hpp*(∞) is the linewidth expected at a high enough temperature for the DC susceptibility to follow the Curie–Weiss law. Therefore, the above formula can be rewritten as:

4 $$\Delta H_{PP} (T)T\chi (T) = \Delta H_{PP} (\infty )C$$

As there are no spin-phonon interactions in the conventional PM regime, such a dependence accounts for a constant to temperature independent^2^. Insert of Fig. 3c shows that the *∆H*_*PP*_*(T)Tχ(T)* does not change until the temperature reaches 360 K then increases distinctly. These behaviors indicate that the magnetic clusters have formed and the existence of an ordered spin clusters well above *T*_*C*_.

**Figure 4:**
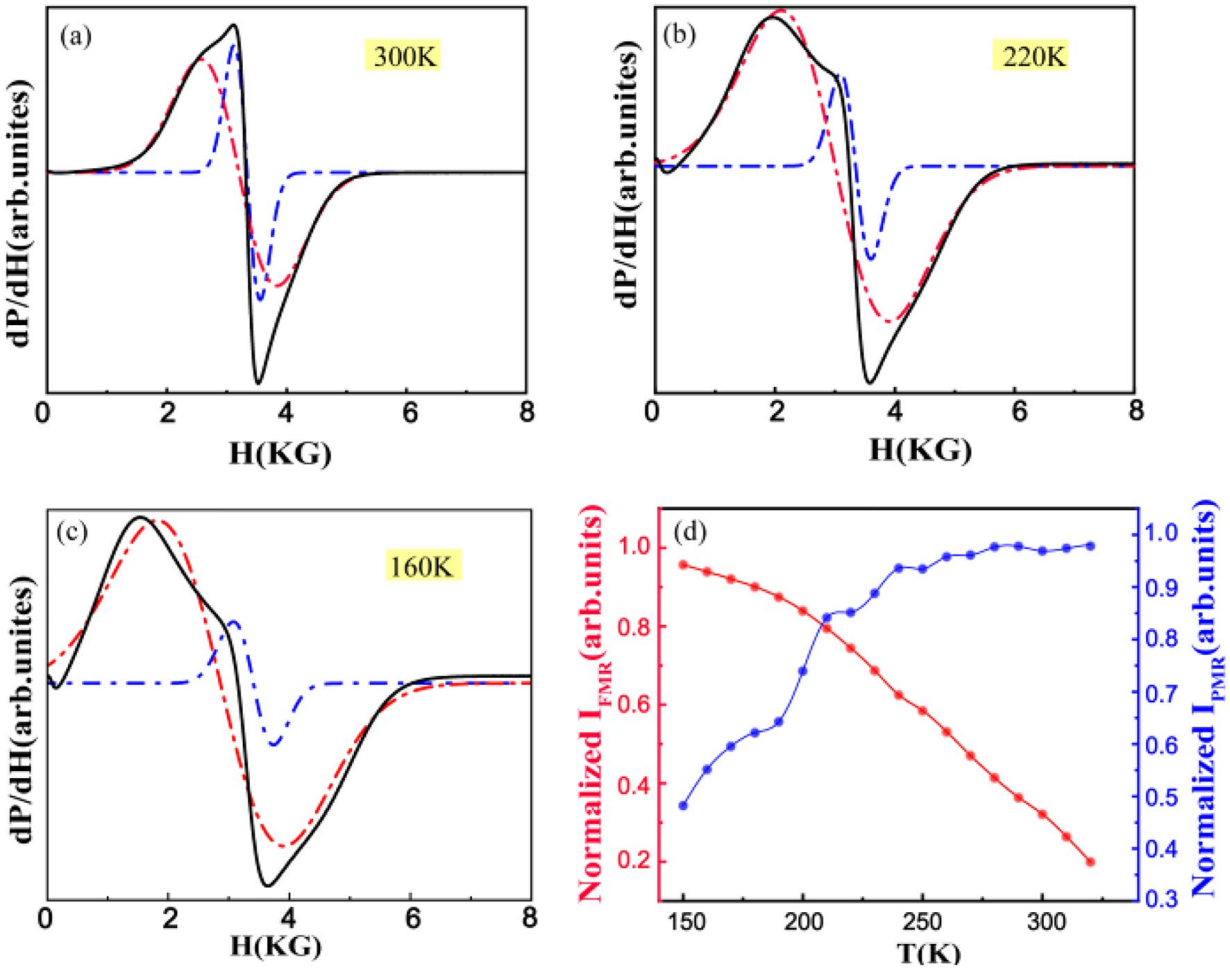
Representative EPR spectra with the fitting spectral dcomposition into PM and FM resonance line at (**a**) 300 K, (**b**) 220 K, (**c**) 160 K. (**d**) The value of IFMR (red line) and IPMR (blue line) from 320 K to 150 K with the fitted PM and PM resonance line.

The data on the normalized the double-integrated intensity (DIN) versus Temperature dependence were presented in Fig. 3d. For *Hr ≫ ∆H*_*PP*_,

5 $$DIN \propto M(T,Hr(T)Hr(T) = \chi \bot (T,Hr(T)),$$

where *M(T, H)* and *χ*_⊥_(*T*, *H*) are the thermodynamic magnetization and transverse magnetic susceptibility^34^. Equation (5) shows that the DIN is only equivalent to magnetic susceptibility when *H*_*r*_ is constant and/or *χ*_⊥_(*T*,*H*_*r*_*(T)*) is field independent. In the pure PM region the DIN can definitely be treated as EPR measured PM susceptibility. In the insert of Fig. 4d, the DIN^*−*1^ versus *T* curve is essentially non-linear, which is similar to the *χ*^*−*1^ versus *T* in Fig. 1a. The CW like regime is observed at 360 K far above *T*_*C*_, and a deviation from the straight line in high temperature is noticeable.

As show in Fig. 2, when the temperature decreases to the *T*_*C*_, a low-field FM response mode emerges gradually at *T* < 320 K, coexisting with the PM resonance line at *g∼*2^14^. As the temperature reduces to *T*_*C*_, the spectral intensity is continuously transferred from the PM resonance to the FM resonance line, but the PM resonance isn’t diminished until the temperature is below *T*_*C*_. To extract the relative spectral weight of each component, the central PM lines can be fitted to a Lorentz lines over the appropriate magnetic-field range around *g∼*2 plus a polynomial background to account for the FMR Contribution^14^. Figure 4 presents the representative EPR spectra with the fitting spectral decomposition into the PM and FM resonance line.

The fitted PM line was subtracted from the total EPR spectra to obtain the FMR component at several significant temperatures of *T*_*1*_ = 300 K, *T*_*2*_ = 220 K and *T*_*3*_ = 160 K in Fig. 4a, b, c. These three temperatures represent several significant temperature intervals during the phase transition, *T*_*3*_ < *T*_*C*_ < *T*_*2*_ < *T*_*platfeatu1*_(280 K) < *T*_*1*_ < *T*_*CW*_, which can be observed clearly from the the plots of d*M*/d*T vs T* (insert of Fig. 1). This phenomenon resulted in a fairly accurate decomposition of the EPR spectra, especially for nanosize magnetic oxide, where the FM resonance line narrows and shifts to a lower magnetic field due to partial orientation effects that effectively increase the splitting of the two signals. Figure 4d shows the value of *I*_*FMR*_ (red line) and *I*_*PMR*_ (blue line) from 320 to 150 K with the fitted PM and FM resonance line. It was found that the value of *I*_*FMR*_ increases gradually when the temperature decreases from 320 K indicating the development and ordering of FM microdomain in the EPR region. Obviously, at 320 K the FM resonance is weak, and this phenomenon is unlike the results obtained from the DC magnetic measurement in Fig. 1a, in which the loop has shown a distinct FM behavior at *T* = 350 K. The reason for that could be induced that the FM signal is too weak for fitting with the Lorentz formula.

## Discussions

**Figure 5:**
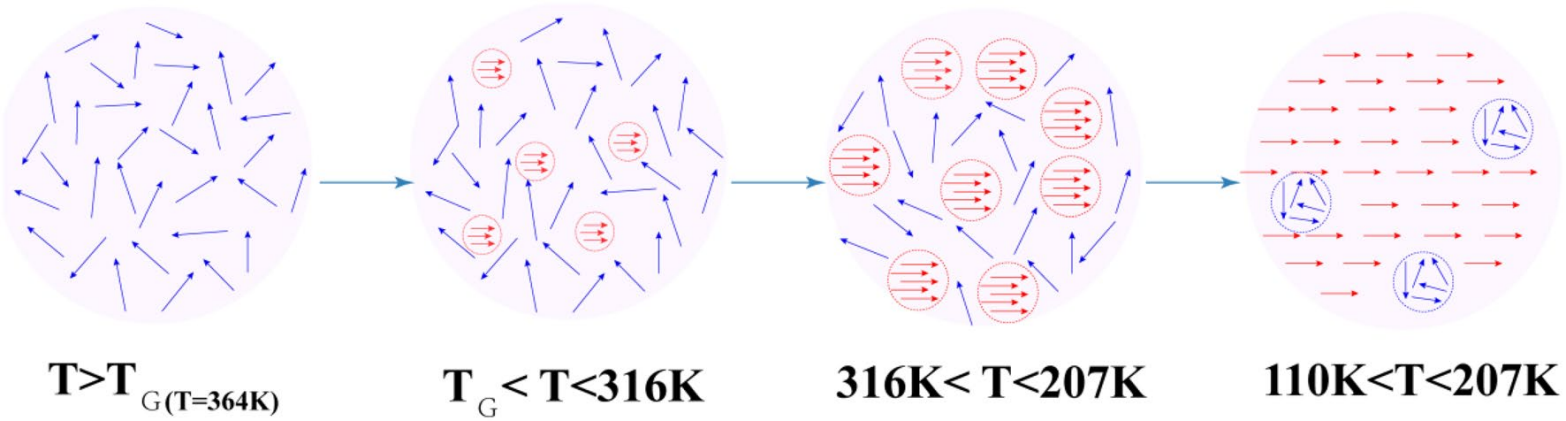
A schematic diagram of the proposed model to describe the magnetic state of La_0.88_Sr_0.12_MnO_3_ nanocrystalline in different temperature regions.

Base on the above argument, a phenomenological scenario was proposed to understand the evolution of the FM phase transition in a LSMO sample. Figure 5 illustrated the model of whole evolution of the magnetic state. At *T* > *T*_*G*_ = 364 K, all the spins are random arrangement and the sample is in the PM state. When the temperature decreases below *T*_*G*_, microdomain/clusters spin disorder generates some FM interactions but these FM clusters are isolated and cannot form a spontaneous magnetization in the material, so the magnetic hysteresis loop presents a weak inflection as shown in Fig. 1c. With a further decrease of temperature from *T*_*θ*_ = 316 K to *T*_*C*_ = 207 K, the FM clusters become larger and more, but they abundant still isolated. Therefore, the FM clusters cannot generate magnetic correlation among themselves. When the temperature decreases to 207 K, most of the spin rotate to the same direction as the extra added magnetic field, but a few spin remain in random arrangement so that some PM clusters remain in the FM state. Comparing with other perovskite manganites, their PM-FM phase transitions are basically no-relaxation. But for the present LSMO nanocrystalline sample, the PM and FM phase coexisted in one regime. These series phenomenon can be attributed to the increasing surface spin in nanocrystalline which weakens the magnetic coupling among domains, similar to the doping method to break the long-range FM interaction. Thus, the localized FM state can appear far above the Curie temperature.

## Conclusions

In conclusion, we have experimentally studied an evolution of the magnetic structural phase generated in LSMO nanocrystalline by the measurements of EPR and DC susceptibility. Based on the variation of g-factor, linewith ∆*H*_*PP*_, and DIN, we found that the linewidth of the EPR signal has a minimum in the vicinity of *T* ∼ *T*_*G*_. At temperatures above *T*_*G*_, the EPR line linearly broadens. The *g*-factor and DIN also have a variation around *T*_*G*_. These EPR characteristics are consistent with the results from the DC susceptibility measurements. We confirmed that a Griffiths-like phase existed at high temperatures far above *T*_*C*_. The formation of a Griffiths phase is due to the short FM ordering state established in the PM regime by increasing the disordering of surface spin.

### Supplementary Information

Supplementary Information.

## Data Availability

The datasets used during the current study available from the corresponding author on reasonable request.
