# Supplementary material for: Evolution of ferromagnetic cluster in perovskite La0.88Sr0.12MnO3 nanocrystalline detected by EPR spectrum
Source: Sci Rep. 2024 Jun 5;14:12898. doi: 10.1038/s41598-024-63512-y (PMC11153607; doi:10.1038/s41598-024-63512-y)
Supplement: Supplementary file 1 — Supplementary Information. [file 41598_2024_63512_MOESM1_ESM.docx]

The structure and phase purity of La_0.88_Sr_0.12_MnO_3_ were examined by XRD measurement in Fig. 1s. The Rietveld method is used to obtain the structural refinement of the XRD data in Fig. 1s. The XRD patterns proved that the sample was pure and a single-phase of orthorhombic structure with space group *Pnma*. Based on the Rietveld refinements, the lattice parameters *a*, *b*, and *c* are found to be 5.467Å, 7.767Å, and 5.537Å, respectively. Refinement of the XRD data gives the following reliability factors: the weighted factor *R_wp_* = 10.06% and *R_p_* = 6.66%, the goodness of fit χ^2^ = 0.9363. Therefore, the fitting results in Fig. 1s. The XRD peaks are broad with large full width at half maximum(FWHM), indicating the formation of LSMO nanocrystalline.


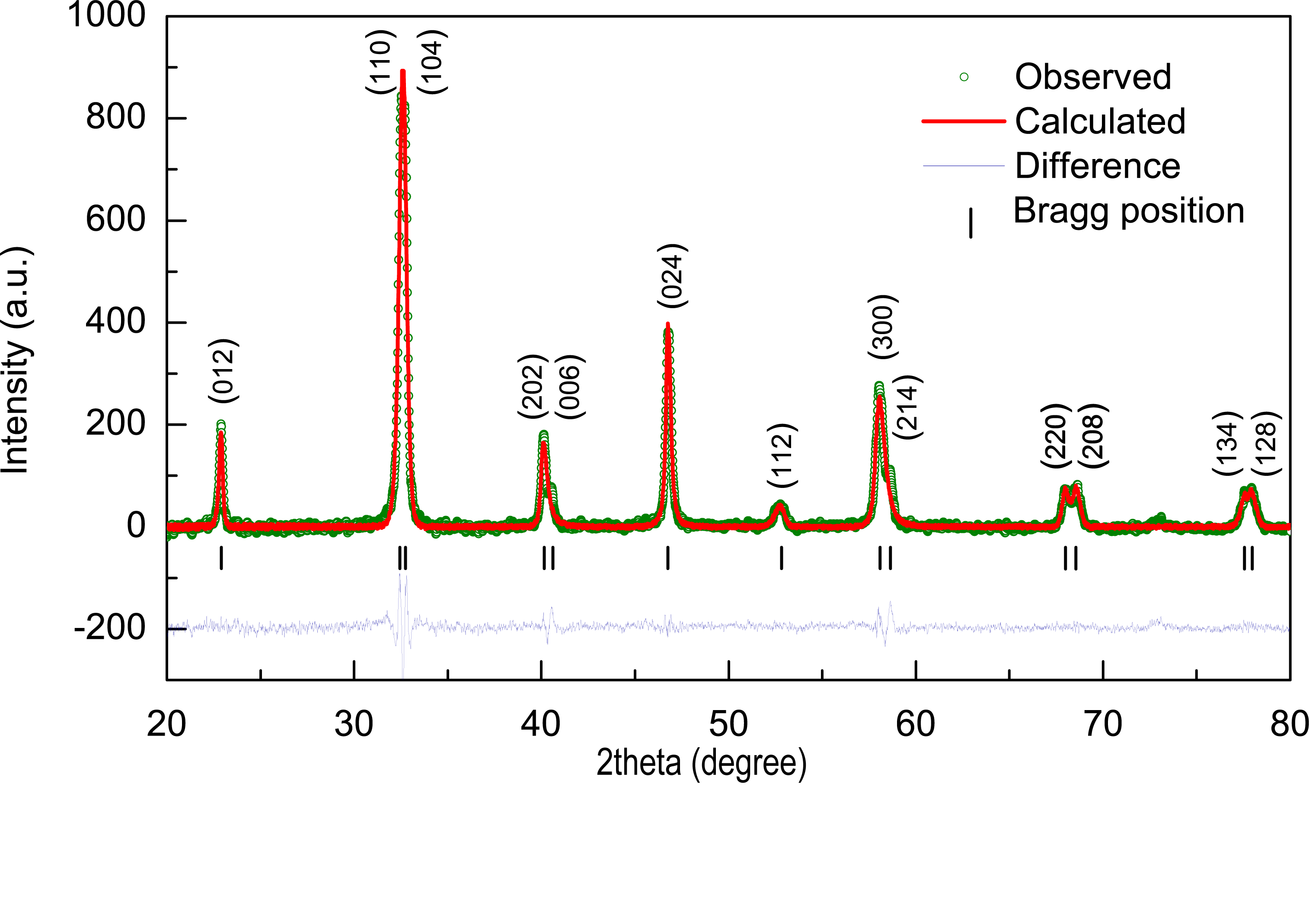


Fig.1s XRD powder diffraction patterns of La_0.88_Sr_0.12_MnO_3_ particles

The size of LSMO particles is homogeneous and the average diameter is about 30 nm estimated through FESEM in Fig.2S.


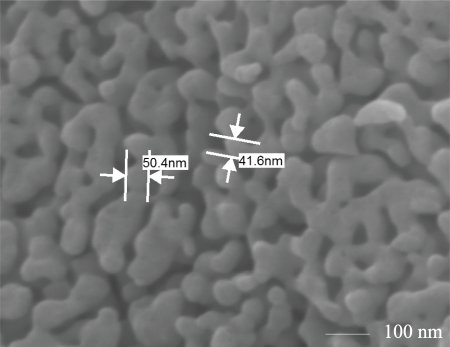


Fig. 2s SEM graph of LSMO particles
